# Pretreatment with ibrutinib reduces cytokine secretion and limits the risk of obinutuzumab-induced infusion-related reactions in patients with CLL: analysis from the iLLUMINATE study

**DOI:** 10.1007/s00277-021-04536-6

**Published:** 2021-05-20

**Authors:** Richard Greil, Alessandra Tedeschi, Carol Moreno, Bertrand Anz, Loree Larratt, Martin Simkovic, Devinder Gill, John G. Gribben, Ian W. Flinn, Zhengyuan Wang, Leo W. K. Cheung, Aaron N. Nguyen, Cathy Zhou, Lori Styles, Fatih Demirkan

**Affiliations:** 1 IIIrd Medical Department Paracelsus Medical University, Salzburg Cancer Research Institute-CCCIT, Cancer Cluster Salzburg, Salzburg, Austria; 2 Department of Hematology, ASST Grande Ospedale Metropolitano Niguarda, Milano, Italy; 3 Department of Hematology, Hospital de la Santa Creu i Sant Pau, Autonomous University of Barcelona, Barcelona, Spain; 4 Department of Medical Oncology, Tennessee Oncology, Chattanooga, TN USA; 5 Department of Clinical Hematology, University of Alberta Hospital, Edmonton, Alberta Canada; 6 Department of Internal Medicine – Hematology, University Hospital and Medical School Hradec Králové, Hradec Králové, Czech Republic; 7 Department of Clinical Hematology, Princess Alexandra Hospital, Brisbane, Queensland Australia; 8 Centre for Haemato-Oncology, Queen Mary University of London, Barts Cancer Institute, London, UK; 9 Center for Blood Cancers, Medical Oncology, Sarah Cannon Research Institute/Tennessee Oncology, Nashville, TN USA; 10 Translational Medicine, Pharmacyclics LLC, an AbbVie Company, Sunnyvale, CA USA; 11 Biostatistics, Pharmacyclics LLC, an AbbVie Company, Sunnyvale, CA USA; 12 Clinical Science, Pharmacyclics LLC, an AbbVie Company, Sunnyvale, CA USA; 13 Department of Hematology, Dokuz Eylül University, Izmir, Turkey

**Keywords:** Infusion-related reactions, Ibrutinib, Obinutuzumab, Cytokines

## Abstract

Anti-CD20 antibody treatments, such as obinutuzumab, have been associated with infusion-related reactions (IRRs). In the phase 3 iLLUMINATE study of ibrutinib-obinutuzumab versus chlorambucil-obinutuzumab in first-line chronic lymphocytic leukemia/small lymphocytic lymphoma, IRRs were substantially reduced with ibrutinib-obinutuzumab versus chlorambucil-obinutuzumab. We prospectively analyzed inflammatory cytokines to evaluate the impact of ibrutinib on circulating cytokine levels following obinutuzumab infusion. In iLLUMINATE, ibrutinib or chlorambucil was given approximately 30–120 min before the first obinutuzumab infusion. Cytokines evaluated were IFNγ, IL-6, IL-8, IL-10, IL-18, MCP-1, MIP-1α, MIP-1β, and TNFα. Changes in peak cytokine levels from baseline (immediately before obinutuzumab) to post-obinutuzumab infusion were compared between arms and between patients with versus without IRRs using Wilcoxon rank sum test. Of 228 treated patients, 95 on ibrutinib-obinutuzumab (15 with IRRs, 80 without) and 88 on chlorambucil-obinutuzumab (45 with IRRs, 43 without) with cytokine data were included. Irrespective of IRR occurrence, median increase in cytokines was lower with ibrutinib-obinutuzumab versus chlorambucil-obinutuzumab for all cytokines (*P* < 0.01) except MIP-1β. Across treatment arms, post-obinutuzumab median increase in all cytokines except MIP-1β was greater in patients with versus without IRRs (*P* < 0.001). IL-6 and IL-8 elevations were associated with IRRs in both treatment arms. Among patients with IRRs, those receiving ibrutinib-obinutuzumab had lower post-obinutuzumab increases in IL-6, IL-8, IL-10, and MCP-1 (*P* < 0.04) than patients receiving chlorambucil-obinutuzumab. For patients in the ibrutinib-treatment arm, we observed a reduction in both the rate of clinically apparent IRRs and the levels of IRR-related cytokines and chemokines. This observation supports an immunomodulatory mechanism of action for ibrutinib. Clinical Trial Registration: NCT02264574

**Supplementary Information:**

The online version contains supplementary material available at 10.1007/s00277-021-04536-6.

## Introduction

Established therapies for the first-line treatment of chronic lymphocytic leukemia (CLL) include novel targeted agents, such as ibrutinib, and chemoimmunotherapy regimens. Typically, chemoimmunotherapy regimens contain anti-CD20 monoclonal antibodies (mAbs), such as rituximab or obinutuzumab. Anti-CD20 mAbs have demonstrated efficacy when used alone or in combination for patients with previously untreated and relapsed/refractory CLL [1–3], with greater efficacy observed for obinutuzumab compared with rituximab when combined with chlorambucil in first-line CLL [3]. Ibrutinib is the only once-daily oral inhibitor of Bruton’s tyrosine kinase (BTK) approved for treatment of CLL in the United States and European Union that has demonstrated significant progression-free survival (PFS) and overall survival (OS) benefit versus established therapies (including chemoimmunotherapy regimens) in randomized phase 3 studies in first-line CLL/small lymphocytic lymphoma (SLL) [2, 4, 5].

Despite their efficacy, the administration of mAbs can result in adverse events including infusion-related reactions (IRRs). IRRs are potentially serious complications that occur rapidly post-infusion and arise from an immune-mediated reaction to the infusion of a drug, notably including mAbs [6]. The severity of IRRs ranges from mild reactions involving flu-like symptoms to rare fatal events.

The rate and severity of IRRs vary depending on the specific treatment. For instance, obinutuzumab is generally associated with more frequent and more severe IRRs than rituximab as observed in the CLL11 study of patients with previously untreated CLL (any grade: 66% versus 38%; grade ≥ 3: 20% versus 4%, respectively) [3] and in the GALLIUM study of patients with follicular lymphoma (any grade: 68% versus 59%; grade ≥ 3: 12% versus 7%, respectively) [3, 7]. A study of patients with relapsed/refractory non-Hodgkin lymphoma who were treated with obinutuzumab resulted in a similar rate of IRRs (any grade: 69%; grade ≥ 3: 11%) [8]. It has been hypothesized that the structure of obinutuzumab (a type II, glycoengineered, humanized mAb) induces greater cellular activation, thereby eliciting the increase in IRRs seen in these studies [9].

In the phase 3 iLLUMINATE (PCYC-1130) study that compared first-line ibrutinib-obinutuzumab and chlorambucil-obinutuzumab in patients with previously untreated CLL, ibrutinib-obinutuzumab demonstrated superior PFS including in patients with one or more high-risk genomic features (del[17p]/*TP53* mutation, del[11q], and/or unmutated *IGHV*) [1]. Of note, the rate of IRRs in iLLUMINATE was lower in patients treated with ibrutinib-obinutuzumab compared with those treated with chlorambucil-obinutuzumab (any grade: 25% versus 58%; grade ≥ 3 or serious: 3% versus 9%, respectively) [1]. This finding supported the notion that the administration of ibrutinib prior to obinutuzumab infusion reduced the risk of IRRs. To investigate the role of ibrutinib in IRRs, we prospectively analyzed patient samples from iLLUMINATE for circulating cytokines and chemokines thought to be associated with IRRs.

## Methods

### Study design and population

iLLUMINATE (PCYC-1130; NCT02264574) is a phase 3, international, randomized open-label study of ibrutinib plus obinutuzumab versus chlorambucil plus obinutuzumab in first-line CLL/SLL. Eligible patients were aged ≥ 18 years with previously untreated, active CLL/SLL and were aged ≥ 65 years or < 65 years with coexisting conditions (Cumulative Illness Rating Scale score > 6, creatinine clearance < 70 mL/min, presence of del(17p) by fluorescent in situ hybridization, or *TP53* mutation). Informed consent was obtained from all participants included in the study. The study design has been previously described [1].

### Procedures

Patients were randomly assigned (1:1) to receive ibrutinib-obinutuzumab or chlorambucil-obinutuzumab. In the ibrutinib-obinutuzumab arm, patients received 420-mg ibrutinib once daily until progressive disease or unacceptable toxicity in combination with obinutuzumab (100 mg on day 1, 900 mg on day 2, 1000 mg on days 8 and 15 of cycle 1, and then 1000 mg on day 1 of each subsequent 28-day cycle for a total of 6 cycles). In the chlorambucil-obinutuzumab arm, patients received 0.5-mg/kg chlorambucil on days 1 and 15 of each 28-day cycle for 6 cycles with obinutuzumab, as above (total of 6 cycles).

All patients received the same, standard prophylactic medications for obinutuzumab-related IRRs that included intravenous corticosteroids, oral analgesics/antipyretics, and an antihistamine. Based on the hypothesis that ibrutinib might reduce the incidence of IRRs, the oral study drug (ibrutinib or chlorambucil) was administered approximately 30 to 120 min before the first obinutuzumab infusion.

Plasma samples were collected at 4 time points on day 1: before the ibrutinib/chlorambucil dose (Online Resource Table S1), immediately prior to infusion of obinutuzumab, and 2 and 4 h into the obinutuzumab infusion. Baseline was defined as immediately before obinutuzumab infusion rather than prior to ibrutinib/chlorambucil treatment, as there were no meaningful differences between the two time points (Online Resource Table S2). Cytokines and chemokines that were evaluated included interferon-γ (IFNγ), interleukin (IL)-6, IL-8/CXCL8, IL-10, IL-18, monocyte chemoattractant protein-1 (MCP-1)/CCL2, macrophage inflammatory protein (MIP)-1α/CCL3, MIP-1β/CCL4, and tumor necrosis factor-α (TNFα). A multi-analyte immunoassay panel (Myriad RBM, Austin, Texas, USA) was used to analyze the level of plasma cytokines and chemokines.

### Statistical analysis

Changes from baseline to post-obinutuzumab infusion peak cytokine/chemokine levels were compared between arms, as well as between patients with and without IRRs, using Wilcoxon rank sum test. A 2-sided *P* value of < 0.05 was considered significant with no adjustments for multiplicity.

Analysis of covariance (ANCOVA) was performed to evaluate each of the baseline factors associated with increase in post-obinutuzumab cytokine levels (log-adjusted) with baseline of the cytokine level as a covariate in the model: sex (male versus female), bulky disease (lymph nodes < 5 cm versus ≥ 5 cm), Rai stage (stage III/IV versus others), splenomegaly (yes/no), age (continuous variable), and log-transformed hematologic parameters (hemoglobin, platelet counts, absolute neutrophil count, and absolute lymphocyte count, all as continuous variables).

Multivariable logistic regression was performed to identify predictors of IRRs. Explanatory variables were first selected based on a univariate logistic regression model at a *P* < 0.2 level among the following factors: treatment arm, baseline clinical factors (stated above), and log-transformed baseline cytokine levels (IFNγ, IL-6, IL-8, IL-10, IL-18, TNFα, MCP-1, MIP-1α, and MIP-1β). Correlation between selected factors was also examined. Independent variables (absolute value of Pearson correlation coefficient < 0.2) were included in the multivariable model. If any pair of the variables correlated, only the variable with the smaller *P* value in the univariate model was entered into the multivariable model.

### Data sharing statement

Requests for access to individual participant data from clinical studies conducted by Pharmacyclics LLC, an AbbVie Company, can be submitted through the Yale Open Data Access (YODA) Project site at http://yoda.yale.edu.

## Results

### Analysis population and patient characteristics

Of 228 treated patients, 95 of 113 patients (84%) in the ibrutinib-obinutuzumab arm and 88 of 115 patients (77%) in the chlorambucil-obinutuzumab arm had cytokine/chemokine results and were included in the cytokine/chemokine analysis population. In the ibrutinib-obinutuzumab arm (*n* = 95), 15 patients (16%) had any grade IRRs on day 1, and 80 patients (84%) did not experience IRRs. In the chlorambucil-obinutuzumab arm (*n* = 88), 45 patients (51%) had any grade IRRs on day 1, and 43 patients (49%) did not experience IRRs.

Baseline characteristics were generally similar between treatment arms in the analysis population (Table 1). Numerically lower platelet counts were observed in the ibrutinib-obinutuzumab arm compared with the chlorambucil-obinutuzumab arm (median, 150 × 10^9^/L versus 162 × 10^9^/L, *P =* 0.0413), which was not a clinically relevant difference. Baseline MCP-1 levels were also significantly lower in the ibrutinib-obinutuzumab arm compared with the chlorambucil-obinutuzumab arm (median, 127 pg/mL versus 191.5 pg/mL, *P =* 0.0089), though both of these values fall within a normal range [10, 11]. Additionally, since a similar arm difference was observed prior to administration of any study drug (Online Resource Table S1), this indicates that the difference was due to the chance of randomization and was not related to any study treatment. Patients in both arms had median baseline levels of IFNγ and IL-6 that were below the detectable threshold of the assay (Table 1), which precluded their inclusion in the analysis as a predictor for the development of IRRs.

**Table 1:** Patient demographics and baseline characteristics in the cytokine analysis population

Characteristic	Ibrutinib-obinutuzumab *n* = 95a	Chlorambucil-obinutuzumab *n* = 88b
Median age, years (range)	70 (47–87)	72 (40–86)
Male, *n* (%)	60 (63)	63 (72)
Rai stage III/IV, *n* (%)	49 (52)	45 (51)
Bulky disease ≥ 5 cm, *n* (%)	25 (26)	33 (38)
Splenomegaly, *n* (%)	80 (84)	74 (84)
Median hemoglobin, g/dL (range)	115 (58–163)	112 (72–153)
Median platelet counts, × 10^9^/L (range)	150 (48–359)	162 (48–463)
Median absolute neutrophil count, × 10^9^/L (range)	6 (0–362)	5 (0–61)
Median absolute lymphocyte count, × 10^9^/L (range)	54 (1–350)	48 (1–386)
Median baseline cytokine levels, pg/mL (range)		
IFNγ	< 6.8 (NA)	< 6.8 (NA)
IL-6	< 3.4 (NA–957)	< 3.4 (NA–252)
IL-8	6.6 (6.0–750)	9.0 (6.0–306)
IL-10	9.0 (5.2–1090)	11.0 (5.2–155)
IL-18	416.0 (69–5540)	401.0 (117–3010)
MCP-1	127.0 (110–1920)	191.5 (110–3090)
MIP-1α	34.0 (25–4070)	30.0 (25–741)
MIP-1β	384.0 (45–44400)	446.0 (45–5920)
TNFα	26.0 (18–887)	24.5 (18–335)

^a^Enrolled in iLLUMINATE, *n* = 113

^b^Enrolled in iLLUMINATE, *n* = 116

*IFN*, interferon; *IL*, interleukin; *MCP*, monocyte chemoattractant protein; *MIP*, macrophage inflammatory protein; *NA*, not available (below the lower limit of detection); *TNF*, tumor necrosis factor

### Analysis of change in cytokine levels by treatment arm and IRR status

**Fig. 1:**
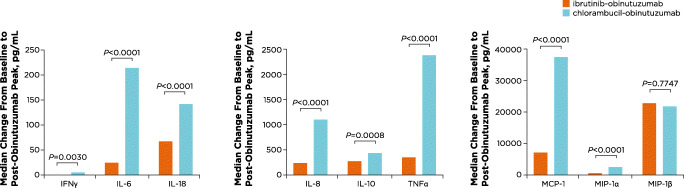
Median changes in cytokine and chemokine levels from baseline to post-obinutuzumab peak by treatment arm. Median changes over baseline (i.e., immediately before obinutuzumab) are shown for all cytokines and chemokines examined by treatment arm. IFN, interferon; IL, interleukin; MCP, monocyte chemoattractant protein; MIP, macrophage inflammatory protein; TNF, tumor necrosis factor

Overall, increases over baseline levels were observed post-obinutuzumab infusion across all cytokines and chemokines analyzed regardless of treatment arm or IRR occurrence. Median change from baseline levels was significantly lower (*P* < 0.01) in patients treated with ibrutinib-obinutuzumab compared with chlorambucil-obinutuzumab for IFNγ, IL-6, IL-18, IL-8, IL-10, TNFα, MCP-1, and MIP-1α (Fig. 1). MIP-1β was the only chemokine for which the change from baseline levels was not significantly different between treatment arms (*P =* 0.7747).

**Fig. 2:**
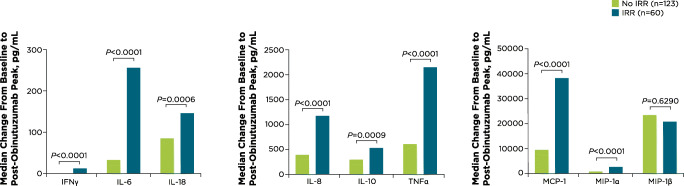
Median changes in cytokine and chemokine levels from baseline to post-obinutuzumab peak by occurrence of IRR on day 1. Median changes over baseline (i.e., immediately before obinutuzumab) are shown for all cytokines and chemokines examined by all patients and their IRR status. IFN, interferon; IL, interleukin; IRR, infusion-related reaction; MCP, monocyte chemoattractant protein; MIP, macrophage inflammatory protein; TNF, tumor necrosis factor

Across both treatment arms, increases in median changes from baseline levels were significantly greater (*P* < 0.001) for patients with IRRs (*n* = 60) compared with patients without IRRs (*n* = 123) for all cytokines and chemokines analyzed except for MIP-1β (*P =* 0.6290) (Fig. 2, Online Resource Fig. S1).

**Fig. 3:**
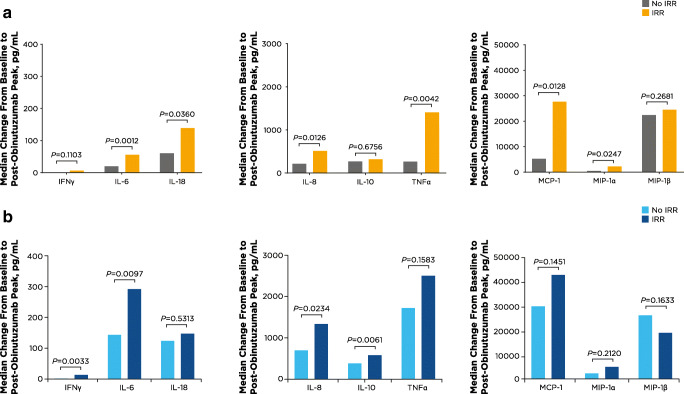
Median changes in cytokine and chemokine levels from baseline to post-obinutuzumab peak by occurrence of IRR on day 1. Median changes over baseline (i.e., immediately before obinutuzumab) are shown for all cytokines and chemokines examined by treatment arm (**a** ibrutinib-obinutuzumab; **b** chlorambucil-obinutuzumab) and IRR occurrence. IFN, interferon; IL, interleukin; IRR, infusion-related reaction; MCP, monocyte chemoattractant protein; MIP, macrophage inflammatory protein; TNF, tumor necrosis factor

Analysis of cytokine/chemokine patterns in patients within each treatment arm showed that greater increases from baseline levels in IL-6 and IL-8 post-obinutuzumab were significantly associated with IRRs in both ibrutinib-obinutuzumab (*P =* 0.0012 and *P =* 0.0126, respectively) and chlorambucil-obinutuzumab treated patients (*P =* 0.0097 and *P =* 0.0234, respectively). In the ibrutinib-obinutuzumab arm, patients with IRRs also had significantly greater increases in post-obinutuzumab levels of IL-18, TNFα, MCP-1, and MIP-1α (*P* < 0.04) (Fig. 3) than patients without IRRs. In the chlorambucil-obinutuzumab arm, patients with IRRs also had greater increases than patients without IRRs in post-obinutuzumab levels of IFNγ and IL-10 (*P* < 0.03) (Fig. 3).

ANCOVA was performed to evaluate potential baseline characteristics associated with the increase in absolute values from baseline of cytokine and chemokine levels observed post-obinutuzumab infusion in each treatment arm. In the ibrutinib-obinutuzumab arm, factors associated with an increase in cytokine and chemokine levels included splenomegaly (IL-6, IL-8, MCP-1, MIP-1α, TNFα), lower hemoglobin levels (IL-8), higher absolute neutrophil count (IL-8, MCP-1, MIP-1α, TNFα), and higher absolute lymphocyte count (IFNγ, IL-6, IL-8, MCP-1, MIP-1α, TNFα). In the chlorambucil-obinutuzumab arm, factors associated with an increase in cytokine and chemokine levels included Rai stage III/IV (IL-8, IL-18), bulky disease (≥ 5 cm: IL-10, TNFα), splenomegaly (IFNγ, IL-18, IL-6, IL-8, MIP-1α, TNFα), lower hemoglobin levels (IL-10, IL-8), lower platelet count (IL-18, MCP-1, MIP-1β), and higher absolute lymphocyte count (IFNγ, IL-10, IL-6, IL-8, MCP-1, MIP-1α, TNFα) (Online Resource Table S3).

**Fig. 4:**
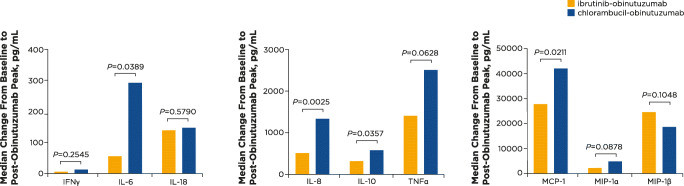
Median changes in cytokine and chemokine levels from baseline to post-obinutuzumab peak by treatment arm in patients with IRRs on day 1. Median changes over baseline (i.e., immediately before obinutuzumab) are shown for all cytokines and chemokines examined by treatment arm for only patients who experienced an IRR. IFN, interferon; IL, interleukin; MCP, monocyte chemoattractant protein; MIP, macrophage inflammatory protein; TNF, tumor necrosis factor

In patients with IRRs and comparing between treatment arms, patients treated with ibrutinib-obinutuzumab had lower increases over baseline in IL-6, IL-8, IL-10, and MCP-1 compared with patients treated with chlorambucil-obinutuzumab (*P* < 0.04) (Fig. 4).

### Multivariable analysis of baseline predictors of IRRs

Among all baseline factors, five were selected as explanatory variables for the multivariable logistic model: treatment arm, sex, absolute lymphocyte count, IL-18, and MCP-1. IFNγ and IL-6 had undetectable levels at baseline and could not be included in the evaluation of IRR predictors. Two baseline factors had a significance of *P* < 0.2 in the univariate model but were excluded due to correlation: baseline bulky disease correlated with baseline absolute lymphocyte count (Pearson correlation coefficient 0.200) and baseline IL-10 correlated with both baseline IL-18 (Pearson coefficient 0.592) and MCP-1 (Pearson coefficient 0.284). All other baseline factors were excluded due to *P* > 0.2 in the univariate analysis. In the final multivariable model, treatment arm, baseline absolute lymphocyte count, and baseline IL-18 were independently associated with the likelihood of IRRs (*P* < 0.05). Within each arm, the multivariable model demonstrated that baseline IL-18 was associated with the likelihood of IRRs in the ibrutinib-obinutuzumab arm, and baseline absolute lymphocyte count was associated with the likelihood of IRRs in the chlorambucil-obinutuzumab arm.

## Discussion

In iLLUMINATE, the frequency and severity of IRRs were reduced in patients treated with ibrutinib-obinutuzumab compared with those treated with chlorambucil-obinutuzumab [1]. This decrease in IRRs with ibrutinib-based treatment is consistent with results from the phase 3 iNNOVATE study, which evaluated ibrutinib combined with rituximab versus rituximab alone in patients with Waldenström’s macroglobulinemia, and showed that any grade and grade ≥ 3 IRRs occurred less frequently with ibrutinib-rituximab versus placebo-rituximab (any grade: 43% versus 59%; grade ≥ 3: 1% versus 12%, respectively) [12]. In a recent phase 1/2 study of ibrutinib-obinutuzumab in first-line CLL, Velez Lujan et al. reported that only 19% of treated patients developed IRR symptoms (grades 1–2: 15%; grade 3: 3%) [13], which compares favorably with historical rates of IRRs with obinutuzumab as single-agent or in other (non-ibrutinib) combination regimens (all grades: 65–90%; grade ≥ 3: 2.5–20%) [3, 14, 15]. Ibrutinib has also been shown to reduce the severity of cytokine release syndrome (CRS) in recent studies of patients with relapsed/refractory CLL receiving chimeric antigen receptor-modified T-cell (CAR-T) therapy [16, 17]. Taken together, these studies indicate a role for ibrutinib in modulating the immune response to certain therapies.

While the mechanism of IRRs is not fully understood, the development of IRRs has been associated with the release of proinflammatory cytokines. In the case of the anti-CD20 mAbs rituximab and obinutuzumab, these proinflammatory cytokines include TNFα, IL-6, and IL-8 [18, 19]. Freeman et al. used multivariable logistic regression models to determine baseline factors associated with IRR risk in the CLL11 study and determined that CD20 expression level was a main predictor of IRRs post-obinutuzumab or rituximab infusion [9]. Additionally, an absolute lymphocyte count of ≥ 50 × 10^9^/L has been shown to be a risk factor for IRRs, particularly for severe IRRs [18].

In a pre-clinical model of CAR-T therapy, the addition of ibrutinib significantly reduced serum cytokines associated with CRS including IFNγ, IL-6, TNFα, IL-2, and GM-CSF [20]. In patients with CLL, treatment with ibrutinib has been shown to decrease regulatory T cells and modulate the cytokine and chemokine milieu in vivo through the decrease of several cytokines (including IL-10, IFNγ, IL-6, and TNFα) and chemokines (including IL-8, MCP-1, MIP-1α, and MIP-1β) [21, 22]. We hypothesized that pretreatment with ibrutinib in patients receiving obinutuzumab infusion led to decreased IRRs because of the potential modulatory effect of ibrutinib on the release of proinflammatory cytokines. In iLLUMINATE, serial measurements of plasma-based inflammatory markers were prospectively performed for various cytokines and chemokines known to be associated with IRRs and anti-CD20 therapy (Online Resource Table S4).

In our analysis, significant increases in post-obinutuzumab levels of IL-6 and IL-8 were observed across both treatment arms in patients with IRRs versus patients who did not experience IRRs. This finding is consistent with prior reports of obinutuzumab-induced IRRs associated with IL-6 and IL-8 [9]. While the post-infusion increase in IL-6 and IL-8 levels among patients with IRRs was common to the 2 arms, the patterns of other cytokine and chemokine levels released post-obinutuzumab differed.

Within the ibrutinib-obinutuzumab arm, patients with IRRs (versus those without IRRs) had significantly higher levels of IL-6, IL-8, TNFα, IL-18, MCP-1, and MIP-1α, whereas in the chlorambucil-obinutuzumab arm, patients with IRRs had significantly higher levels of IFNγ, IL-6, IL-8, and IL-10 compared with those without IRRs. In a multivariable analysis of baseline factors associated with IRRs during ibrutinib-obinutuzumab treatment, only higher baseline levels of IL-18 predicted the risk of developing IRRs. For patients in the chlorambucil-obinutuzumab arm, elevated baseline absolute lymphocyte counts and MCP-1 were factors independently predictive of IRR risk. Results from the multivariable analysis are limited given that some cytokines (particularly IFNγ and IL-6) had baseline levels that were below the detectable threshold of the assay, preventing their inclusion in the multivariable analysis assessing for predictive variables for IRRs. In addition, the small number of patients who experienced IRRs within the ibrutinib-obinutuzumab arm (*n* = 15) may also limit interpretations of the current analysis.

These findings differ from a recent report of cytokine analysis from a phase 1/2 study in 32 patients treated with first-line ibrutinib-obinutuzumab [13]. In that study, significant increases from baseline in IFNγ, MIP-1α, and TNFα post-obinutuzumab infusion were observed in patients who developed IRRs. In univariate analysis, baseline levels of IFNγ, IL-6, and MIP-1α were found to be significantly higher in patients who developed IRRs. Differences between findings from our current analysis and the published phase 1/2 report with ibrutinib-obinutuzumab may be partially due to patient population differences in burden of disease, panels of cytokines and chemokines analyzed (iLLUMINATE included MCP-1), methods of analysis, and sample size (the larger number of patients in iLLUMINATE allowed multivariable analysis).

Our analysis shows that patients treated with ibrutinib versus chlorambucil prior to obinutuzumab infusion had significantly lower increases from baseline for all cytokines and chemokines, except MIP-1β. The pattern of cytokines released during IRRs appeared to differ by treatment regimen, where significantly lower increases in post-obinutuzumab cytokine levels were observed with ibrutinib-obinutuzumab versus chlorambucil-obinutuzumab for IL-6, IL-8, IL-10, and MCP-1. Increases in IL-6 and IL-8 levels have been shown to be related to IRRs induced by obinutuzumab [19]. IL-10 is an anti-inflammatory cytokine that functions to suppress and dampen immune responses. Increases in IL-10 were observed in a patient with diffuse large B-cell lymphoma who experienced a fatal IRR after treatment with rituximab [23]. MCP-1 is a chemokine that acts as a main chemoattractant for monocytes and macrophages, and elevated levels of MCP-1 were associated with severe cytokine release syndrome in patients with B-cell malignancies treated with chimeric antigen receptor T-cell therapy [24].

The mechanism by which ibrutinib treatment decreases or inhibits circulating cytokine and chemokine levels that contribute to IRRs remains unknown. An in vitro study showed that rituximab mediated the CD20 trogocytosis of B cells that led to the rapid production and release of IL-6 [25]. Ibrutinib has been shown to potently inhibit trogocytosis mediated by anti-CD20 mAbs in CLL cells in vitro [26]. This inhibition of trogocytosis by ibrutinib could potentially explain the attenuated release of IL-6 observed in the current analysis; however, further studies are needed to investigate the exact mechanism. Although several pre-clinical studies have shown that treatment with ibrutinib may antagonize the mechanism of anti-CD20 antibodies (particularly as it relates to cell-mediated effector mechanisms such as antibody-dependent cellular cytotoxicity) [27–29], these observations have not translated to the clinical setting [14, 30–32]. In the recent phase 3 Alliance (A041202) study that evaluated ibrutinib-based treatment (single-agent ibrutinib or ibrutinib-rituximab combination) versus bendamustine-rituximab in first-line CLL, slightly higher responses were observed for ibrutinib-rituximab than for single-agent ibrutinib (complete response [CR] rate: 12% and 7%; undetectable minimal residual disease [MRD] 4% and 1%). There was no difference, however, in PFS between the two arms (88% versus 87% at 2 years), although it should be noted the study was not designed to compare between the two ibrutinib-based treatment arms [31].

The current analysis demonstrates that ibrutinib suppresses obinutuzumab-induced increases in multiple cytokines and chemokines related to IRRs and suggests that the reduction in IRR events in ibrutinib-treated patients is the result of a reduction in the release of inflammatory cytokines and chemokines. These observations may have implications for immunotherapeutic approaches where the tuning of the inflammatory reaction may modify the type and degree of cytokine release patterns (activated or dampened) and their role in the treatment effect or safety profile of immunotherapies.

## Supplementary information

ESM 1 (DOCX 105 kb)

## Data Availability

Requests for access to individual participant data from clinical studies conducted by Pharmacyclics LLC, an AbbVie Company, can be submitted through the Yale Open Data Access (YODA) Project site at http://yoda.yale.edu.

## References

[1] Moreno C, Greil R, Demirkan F, Tedeschi A, Anz B, Larratt L, Simkovic M, Samoilova O, Novak J, Ben-Yehuda D, Strugov V, Gill D, Gribben JG, Hsu E, Lih CJ, Zhou C, Clow F, James DF, Styles L, Flinn IW (2019). Ibrutinib plus obinutuzumab versus chlorambucil plus obinutuzumab in first-line treatment of chronic lymphocytic leukaemia (iLLUMINATE): a multicentre, randomised, open-label, phase 3 trial. Lancet Oncol.

[2] Shanafelt TD, Wang XV, Kay NE, Hanson CA, O'Brien S, Barrientos J, Jelinek DF, Braggio E, Leis JF, Zhang CC, Coutre SE, Barr PM, Cashen AF, Mato AR, Singh AK, Mullane MP, Little RF, Erba H, Stone RM, Litzow M, Tallman M (2019). Ibrutinib-rituximab or chemoimmunotherapy for chronic lymphocytic leukemia. N Engl J Med.

[3] Goede V, Fischer K, Busch R, Engelke A, Eichhorst B, Wendtner CM, Chagorova T, de la Serna J, Dilhuydy MS, Illmer T, Opat S, Owen CJ, Samoylova O, Kreuzer KA, Stilgenbauer S, Dohner H, Langerak AW, Ritgen M, Kneba M, Asikanius E, Humphrey K, Wenger M, Hallek M (2014). Obinutuzumab plus chlorambucil in patients with CLL and coexisting conditions. N Engl J Med.

[4] Burger JA, Tedeschi A, Barr PM, Robak T, Owen C, Ghia P, Bairey O, Hillmen P, Bartlett NL, Li J, Simpson D, Grosicki S, Devereux S, McCarthy H, Coutre S, Quach H, Gaidano G, Maslyak Z, Stevens DA, Janssens A, Offner F, Mayer J, O'Dwyer M, Hellmann A, Schuh A, Siddiqi T, Polliack A, Tam CS, Suri D, Cheng M, Clow F, Styles L, James DF, Kipps TJ, RESONATE-2 Investigators (2015). Ibrutinib as initial therapy for patients with chronic lymphocytic leukemia. N Engl J Med.

[5] Byrd JC, Brown JR, O'Brien S, Barrientos JC, Kay NE, Reddy NM, Coutre S, Tam CS, Mulligan SP, Jaeger U, Devereux S, Barr PM, Furman RR, Kipps TJ, Cymbalista F, Pocock C, Thornton P, Caligaris-Cappio F, Robak T, Delgado J, Schuster SJ, Montillo M, Schuh A, de Vos S, Gill D, Bloor A, Dearden C, Moreno C, Jones JJ, Chu AD, Fardis M, McGreivy J, Clow F, James DF, Hillmen P, Investigators RESONATE (2014). Ibrutinib versus ofatumumab in previously treated chronic lymphoid leukemia. N Engl J Med.

[6] Baldo BA (2013). Adverse events to monoclonal antibodies used for cancer therapy: focus on hypersensitivity responses. Oncoimmunology.

[7] Marcus R, Davies A, Ando K, Klapper W, Opat S, Owen C, Phillips E, Sangha R, Schlag R, Seymour JF, Townsend W, Trneny M, Wenger M, Fingerle-Rowson G, Rufibach K, Moore T, Herold M, Hiddemann W (2017). Obinutuzumab for the first-line treatment of follicular lymphoma. N Engl J Med.

[8] Sehn LH, Chua N, Mayer J, Dueck G, Trneny M, Bouabdallah K, Fowler N, Delwail V, Press O, Salles G, Gribben J, Lennard A, Lugtenburg PJ, Dimier N, Wassner-Fritsch E, Fingerle-Rowson G, Cheson BD (2016). Obinutuzumab plus bendamustine versus bendamustine monotherapy in patients with rituximab-refractory indolent non-Hodgkin lymphoma (GADOLIN): a randomised, controlled, open-label, multicentre, phase 3 trial. Lancet Oncol.

[9] Freeman CL, Dixon M, Houghton R, Kreuzer KA, Fingerle-Rowson G, Herling M, Humphrey K, Bottcher S, de Costa CS, Iglesias V, Stilgenbauer S, Gribben J, Hallek M, Goede V (2016). Role of CD20 expression and other pre-treatment risk factors in the development of infusion-related reactions in patients with CLL treated with obinutuzumab. Leukemia.

[10] Guan R, Purohit S, Wang H, Bode B, Reed JC, Steed RD, Anderson SW, Steed L, Hopkins D, Xia C, She JX (2011). Chemokine (C-C motif) ligand 2 (CCL2) in sera of patients with type 1 diabetes and diabetic complications. PLoS One.

[11] Lubowicka E, Przylipiak A, Zajkowska M, Piskor BM, Malinowski P, Fiedorowicz W, Lawicki S (2018). Plasma chemokine CCL2 and its receptor CCR2 concentrations as diagnostic biomarkers for breast cancer patients. Biomed Res Int.

[12] Dimopoulos MA, Tedeschi A, Trotman J, Garcia-Sanz R, Macdonald D, Leblond V, Mahe B, Herbaux C, Tam C, Orsucci L, Palomba ML, Matous JV, Shustik C, Kastritis E, Treon SP, Li J, Salman Z, Graef T, Buske C, iNNOVATE Study Group, European Consortium for Waldenstrom’s Macroglobulinemia (2018). Phase 3 trial of ibrutinib plus rituximab in Waldenstrom's macroglobulinemia. N Engl J Med.

[13] Lujan JV, Lengerke-Diaz PA, Jacobs C, Moreno-Cortes EF, Ramirez-Segura CA, Choi MY, McCarthy C, Heinen A, Kipps TJ, Castro JE (2020). Ibrutinib reduces obinutuzumab infusion-related reactions in patients with chronic lymphocytic leukemia and is associated with changes in plasma cytokine levels. Haematologica.

[14] Byrd JC, Flynn JM, Kipps TJ, Boxer M, Kolibaba KS, Carlile DJ, Fingerle-Rowson G, Tyson N, Hirata J, Sharman JP (2016). Randomized phase 2 study of obinutuzumab monotherapy in symptomatic, previously untreated chronic lymphocytic leukemia. Blood.

[15] Brown JR, O'Brien S, Kingsley CD, Eradat H, Pagel JM, Lymp J, Hirata J, Kipps TJ (2015). Obinutuzumab plus fludarabine/cyclophosphamide or bendamustine in the initial therapy of CLL patients: the phase 1b GALTON trial. Blood.

[16] Gill SI, Vides V, Frey NV, Metzger S, O'Brien M, Hexner E, Mato AR, Lacey SF, Melenhorst JJ, Pequignot E, Gladney WL, Hwang W-T, Lamontagne A, Davis M, Byrd JC, Schuster SJ, Siegel DL, Isaacs RE, June CH, Porter DL (2018). Prospective clinical trial of anti-CD19 CAR T cells in combination with ibrutinib for the treatment of chronic lymphocytic leukemia shows a high response rate (Abstract). Blood.

[17] Gauthier J, Hirayama AV, Hay KA, Li D, Lymp J, Sheih A, Purushe J, Pender BS, Hawkins RM, Vakil A, Phi T-D, Steinmetz RN, Chapuis AG, Till BG, Dhawale T, Hendrie PC, Kiem H-P, Ramos J, Shadman M, Cassaday RD, Acharya UH, Riddell SR, Maloney DG, Turtle CJ (2018). Comparison of efficacy and toxicity of CD19-specific chimeric antigen receptor T-cells alone or in combination with ibrutinib for relapsed and/or refractory CLL (Abstract). Blood.

[18] Winkler U, Jensen M, Manzke O, Schulz H, Diehl V, Engert A (1999). Cytokine-release syndrome in patients with B-cell chronic lymphocytic leukemia and high lymphocyte counts after treatment with an anti-CD20 monoclonal antibody (rituximab, IDEC-C2B8). Blood.

[19] Freeman CL, Morschhauser F, Sehn L, Dixon M, Houghton R, Lamy T, Fingerle-Rowson G, Wassner-Fritsch E, Gribben JG, Hallek M, Salles G, Cartron G (2015). Cytokine release in patients with CLL treated with obinutuzumab and possible relationship with infusion-related reactions. Blood.

[20] Ruella M, Kenderian SS, Shestova O, Klichinsky M, Melenhorst JJ, Wasik MA, Lacey SF, June CH, Gill S (2017). Kinase inhibitor ibrutinib to prevent cytokine-release syndrome after anti-CD19 chimeric antigen receptor T cells for B-cell neoplasms. Leukemia.

[21] Niemann CU, Herman SE, Maric I, Gomez-Rodriguez J, Biancotto A, Chang BY, Martyr S, Stetler-Stevenson M, Yuan CM, Calvo KR, Braylan RC, Valdez J, Lee YS, Wong DH, Jones J, Sun C, Marti GE, Farooqui MZ, Wiestner A (2016). Disruption of in vivo chronic lymphocytic leukemia tumor-microenvironment interactions by ibrutinib – findings from an investigator-initiated phase II study. Clin Cancer Res.

[22] Podhorecka M, Goracy A, Szymczyk A, Kowal M, Ibanez B, Jankowska-Lecka O, Macheta A, Nowaczynska A, Drab-Urbanek E, Chocholska S, Jawniak D, Hus M (2017). Changes in T-cell subpopulations and cytokine network during early period of ibrutinib therapy in chronic lymphocytic leukemia patients: the significant decrease in T regulatory cells number. Oncotarget.

[23] Gutierrez A, Rodriguez J, Martinez J, Amezaga R, Ramos R, Galmes B, Bea MD, Ferrer J, Pons J, Sampol A, Morey M, Duran MA, Raurich J, Besalduch J (2006). Pathogenic study of anti-CD20 infusion-related severe refractory shock in diffuse large B-cell lymphoma. Leuk Lymphoma.

[24] Hay KA, Hanafi LA, Li D, Gust J, Liles WC, Wurfel MM, Lopez JA, Chen J, Chung D, Harju-Baker S, Cherian S, Chen X, Riddell SR, Maloney DG, Turtle CJ (2017). Kinetics and biomarkers of severe cytokine release syndrome after CD19 chimeric antigen receptor-modified T-cell therapy. Blood.

[25] Jones JD, Hamilton BJ, Skopelja S, Rigby WF (2014). Induction of interleukin-6 production by rituximab in human B cells. Arthritis Rheumatol.

[26] Skarzynski M, Niemann CU, Lee YS, Martyr S, Maric I, Salem D, Stetler-Stevenson M, Marti GE, Calvo KR, Yuan C, Valdez J, Soto S, Farooqui MZ, Herman SE, Wiestner A (2016). Interactions between ibrutinib and anti-CD20 antibodies: competing effects on the outcome of combination therapy. Clin Cancer Res.

[27] Kohrt HE, Sagiv-Barfi I, Rafiq S, Herman SE, Butchar JP, Cheney C, Zhang X, Buggy JJ, Muthusamy N, Levy R, Johnson AJ, Byrd JC (2014). Ibrutinib antagonizes rituximab-dependent NK cell-mediated cytotoxicity. Blood.

[28] Da Roit F, Engelberts PJ, Taylor RP, Breij EC, Gritti G, Rambaldi A, Introna M, Parren PW, Beurskens FJ, Golay J (2015). Ibrutinib interferes with the cell-mediated anti-tumor activities of therapeutic CD20 antibodies: implications for combination therapy. Haematologica.

[29] Borge M, Belen Almejun M, Podaza E, Colado A, Fernandez Grecco H, Cabrejo M, Bezares RF, Giordano M, Gamberale R (2015). Ibrutinib impairs the phagocytosis of rituximab-coated leukemic cells from chronic lymphocytic leukemia patients by human macrophages. Haematologica.

[30] Tedeschi A, Greil R, Demirkan F, Robak T, Moreno C, Barr PM, Anz B, Simpson D, Gaidano G, Bairey O, Stevens D, Gill D, Flinn IW, Kipps TJ, Burger JA, Lin J, Webb T, Fedorov V, Styles L, Gribben JG (2020). A cross-trial comparison of single-agent ibrutinib versus chlorambucil-obinutuzumab in previously untreated patients with chronic lymphocytic leukemia or small lymphocytic lymphoma. Haematologica.

[31] Woyach JA, Ruppert AS, Heerema NA, Zhao W, Booth AM, Ding W, Bartlett NL, Brander DM, Barr PM, Rogers KA, Parikh SA, Coutre S, Hurria A, Brown JR, Lozanski G, Blachly JS, Ozer HG, Major-Elechi B, Fruth B, Nattam S, Larson RA, Erba H, Litzow M, Owen C, Kuzma C, Abramson JS, Little RF, Smith SE, Stone RM, Mandrekar SJ, Byrd JC (2018). Ibrutinib regimens versus chemoimmunotherapy in older patients with untreated CLL. N Engl J Med.

[32] Burger JA, Sivina M, Jain N, Kim E, Kadia T, Estrov Z, Nogueras-Gonzalez GM, Huang X, Jorgensen J, Li J, Cheng M, Clow F, Ohanian M, Andreeff M, Mathew T, Thompson P, Kantarjian H, O'Brien S, Wierda WG, Ferrajoli A, Keating MJ (2019). Randomized trial of ibrutinib vs ibrutinib plus rituximab in patients with chronic lymphocytic leukemia. Blood.

