# Supplementary material for: Pretreatment with ibrutinib reduces cytokine secretion and limits the risk of obinutuzumab-induced infusion-related reactions in patients with CLL: analysis from the iLLUMINATE study
Source: Ann Hematol. 2021 May 20;100(7):1733–42. doi: 10.1007/s00277-021-04536-6 (PMC8195966; doi:10.1007/s00277-021-04536-6)
Supplement: Supplementary file 1 — (DOCX 105 kb) [file 277_2021_4536_MOESM1_ESM.docx]

**SUPPLEMENTARY MATERIAL**

**Target Journal**: *Annals of Hematology*

**Pretreatment with ibrutinib reduces cytokine secretion and limits the risk of obinutuzumab-induced infusion-related reactions in patients with CLL: analysis from the iLLUMINATE study**

Richard Greil, MD^1^; Alessandra Tedeschi, MD^2^; Carol Moreno, MD, PhD^3^; Bertrand Anz, MD^4^; Loree Larratt, MD^5^; Martin Simkovic, MD, PhD^6^; Devinder Gill, MD, MRCP, FRCPath^7^; John G. Gribben, MD, DSc, FRCP, FRCPath, Fmed Sci^8^; Ian W. Flinn, MD, PhD^9^; Zhengyuan Wang, PhD^10^; Leo W.K. Cheung, PhD^10^; Aaron N. Nguyen, PhD^10^; Cathy Zhou, MS^11^; Lori Styles, MD^12^; Fatih Demirkan, MD^13^

*^1^IIIrd Medical Department, Paracelsus Medical University, Salzburg Cancer Research Institute-CCCIT, Cancer Cluster Salzburg, Salzburg, Austria; ^2^Department of Hematology, ASST Grande Ospedale Metropolitano Niguarda, Milano, Italy; ^3^Department of Hematology, Hospital de la Santa Creu i Sant Pau, Autonomous University of Barcelona, Barcelona, Spain; ^4^Department of Medical Oncology, Tennessee Oncology, Chattanooga, TN, USA; ^5^Department of Clinical Hematology, University of Alberta Hospital, Edmonton, Alberta, Canada; ^6^Department of Internal Medicine – Hematology, University Hospital and Medical School Hradec Králové, Hradec Králové, Czech Republic; ^7^Department of Clinical Hematology, Princess Alexandra Hospital, Brisbane, Queensland, Australia; ^8^Centre for Haemato-Oncology, Queen Mary, University of London, Barts Cancer Institute, London, UK; ^9^Center for Blood Cancers, Medical Oncology, Sarah Cannon Research Institute/Tennessee Oncology, Nashville, TN, USA; ^10^Translational Medicine, Pharmacyclics LLC, an AbbVie Company, Sunnyvale, CA, USA; ^11^Biostatistics, Pharmacyclics LLC, an AbbVie Company, Sunnyvale, CA, USA; ^12^Clinical Science, Pharmacyclics LLC, an AbbVie Company, Sunnyvale, CA, USA; ^13^Department of Hematology, Dokuz Eylül University, Izmir, Turkey.*

**Correspondence to:**

Richard Greil, MD

E-mail: [r.greil@salk.at](mailto:r.greil@salk.at)

**Table S1. Pre-Dose^a^ Cytokine Levels**

|  | **Ibrutinib- obinutuzumab**  **n=95^b^** | **Chlorambucil- obinutuzumab**  **n=88^c^** |
| --- | --- | --- |
| Median baseline cytokine levels, pg/mL (range) |  |  |
| IFNγ | <6.8 (NA) | <6.8 (NA) |
| IL-6 | <3.4 (NA–940) | <3.4 (NA–334) |
| IL-8 | 7.7 (6.0–477) | 9.2 (6.0–440) |
| IL-10 | 9.5 (5.2–14600) | 10.0 (5.2–148) |
| IL-18 | 409.0 (63–4500) | 394.0 (145–4310) |
| MCP-1 | 145.0 (110–1620) | 185.0 (110–4050) |
| MIP-1α | 35.0 (25–13100) | 50.0 (25–5840) |
| MIP-1β | 469.0 (65–26500) | 576.5 (65–29300) |
| TNFα | 29.0 (18–1070) | 29.0 (18–718) |

^a^Pre-dose defined as last available cytokine value before first dose of ibrutinib or chlorambucil; ^b^Enrolled in iLLUMINATE, n=113; ^c^Enrolled in iLLUMINATE, n=116.

IFN, interferon; IL, interleukin; MCP, monocyte chemoattractant protein; MIP, macrophage inflammatory protein; NA, not available (below the lower limit of detection); TNF, tumor necrosis factor.

**Table S2. Mean Difference of Pre-Infusion^a^ and Pre-Dose^b^ Cytokine Levels**

|  | **Ibrutinib-obinutuzumab**  **n=95^c^** | | **Chlorambucil-obinutuzumab**  **n=88^d^** | |
| --- | --- | --- | --- | --- |
|  | **Mean Difference**  **(95% CI)** | ***P-*value** | **Mean Difference**  **(95% CI)** | ***P-*value** |
| IFNγ | 0 | - | 0 | - |
| IL-6 | -1.7 (-30.0–26.4) | 0.8962 | -4.93 (-10.9–1.1) | 0.1048 |
| IL-8 | 0.1 (-17.5–17.6) | 0.9927 | -10.41 (-25.6–4.8) | 0.1777 |
| IL-10 | -141.5 (-423.9–140.9) | 0.3224 | 2.84 (0.1–5.6) | 0.0445 |
| IL-18 | 6.6 (-43.3–56.6) | 0.7927 | -18.3 (-58.1–21.4) | 0.3615 |
| MCP-1 | -30.3 (-47.8– -12.9) | 0.0008 | -41.1 (-79.8–2.5) | 0.0371 |
| MIP-1α | -87.6 (-373.5–198.3) | 0.5445 | -111.9 (-244.9–21.0) | 0.0979 |
| MIP-1β | 233.7 (-768.9–1236.4) | 0.6445 | -677.1 (-1365.5–11.3) | 0.0538 |
| TNFα | -17.1 (-42.8–8.6) | 0.1904 | -21.1 (-43.9–1.6) | 0.0677 |

^a^Pre-infusion defined as last available cytokine value before obinutuzumab infusion; ^b^Pre-dose defined as last available cytokine value before first dose of ibrutinib or chlorambucil; ^c^Enrolled in iLLUMINATE, n=113; ^d^Enrolled in iLLUMINATE, n=116.

IFN, interferon; IL, interleukin; MCP, monocyte chemoattractant protein; MIP, macrophage inflammatory protein; TNF, tumor necrosis factor.

**Table S3. Baseline Factors Associated With an Increase in Post-Obinutuzumab Cytokines and Chemokines (ANCOVA)**

|  | **Sex  (male/ female)** | **Age** | **Rai stage (III/IV vs Others)** | **Bulky disease (≥5 cm)** | **Splenomegaly (Yes/No)** | **Hemoglobin**^a^ | **Platelet count**^a^ | **Absolute neutrophil count**^a^ | **Absolute lymphocyte count**^a^ |
| --- | --- | --- | --- | --- | --- | --- | --- | --- | --- |
|  | **Chlorambucil-obinutuzumab *P* values** | | | | | | | | |
| IFNγ | 0.24 | 0.4968 | 0.7217 | 0.0694 | 0.0296 | 0.4484 | 0.1371 | 0.9248 | 0.0103 |
| IL-6 | 0.0917 | 0.6407 | 0.056 | 0.0502 | 0.0006 | 0.3873 | 0.3034 | 0.6057 | 0.0083 |
| IL-18 | 0.9648 | 0.6264 | 0.024 | 0.3543 | 0.0482 | 0.1724 | 0.0406 | 0.2909 | 0.0854 |
| IL-8 | 0.304 | 0.1473 | 0.0023 | 0.0801 | 0.0101 | 0.0055 | 0.1491 | 0.6184 | 0.0006 |
| IL-10 | 0.2885 | 0.5081 | 0.0925 | 0.0216 | 0.2854 | 0.0282 | 0.213 | 0.9932 | 0.0112 |
| TNFα | 0.5309 | 0.8695 | 0.0831 | 0.0191 | 0.0044 | 0.2925 | 0.2313 | 0.7768 | 0.0056 |
| MCP-1 | 0.2325 | 0.2538 | 0.1206 | 0.191 | 0.1437 | 0.5193 | 0.0052 | 0.4165 | 0.0447 |
| MIP-1α | 0.0924 | 0.5066 | 0.7918 | 0.1073 | 0.0041 | 0.9248 | 0.3106 | 0.7758 | 0.0194 |
| MIP-1β | 0.9643 | 0.3904 | 0.7024 | 0.7009 | 0.2807 | 0.9507 | 0.0037 | 0.3298 | 0.0522 |
|  | **Ibrutinib-obinutuzumab *P* values** | | | | | | | | |
| IFNγ | 0.1412 | 0.6661 | 0.3542 | 0.8848 | 0.2089 | 0.3778 | 0.5049 | 0.0515 | 0.036 |
| IL-6 | 0.4238 | 0.4879 | 0.912 | 0.4571 | 0.0018 | 0.0947 | 0.5173 | 0.1015 | 0.0039 |
| IL-18 | 0.2224 | 0.5420 | 0.8887 | 0.6864 | 0.5863 | 0.6387 | 0.4131 | 0.4572 | 0.751 |
| IL-8 | 0.7698 | 0.4791 | 0.3519 | 0.3591 | 0.0027 | 0.0016 | 0.5969 | 0.0288 | <0.0001 |
| IL-10 | 0.2141 | 0.6606 | 0.7187 | 0.5845 | 0.9679 | 0.2347 | 0.3738 | 0.3566 | 0.218 |
| TNFα | 0.5854 | 0.7083 | 0.6438 | 0.5237 | 0.0161 | 0.0937 | 0.6336 | 0.0218 | 0.0002 |
| MCP-1 | 0.9513 | 0.9139 | 0.636 | 0.1894 | 0.0049 | 0.1872 | 0.9389 | 0.0126 | 0.0001 |
| MIP-1α | 0.163 | 0.8745 | 0.4573 | 0.5629 | 0.0122 | 0.2341 | 0.9289 | 0.0174 | 0.0003 |
| MIP-1β | 0.5252 | 0.9250 | 0.5819 | 0.1605 | 0.2871 | 0.4596 | 0.5741 | 0.2879 | 0.0529 |

^a^ Log-transformed values were used in the ANCOVA model.

IFN, interferon; IL, interleukin; MCP, monocyte chemoattractant protein; MIP, macrophage inflammatory protein; TNF, tumor necrosis factor.

**Fig. S1. Cytokine and Chemokine Expression by IRR Occurrence and Treatment Arm.** Heat map analysis of post-obinutuzumab peak cytokine expression in individual patients by occurrence of IRR and treatment arm. Clb-G, chlorambucil-obinutuzumab; Ibr-G, ibrutinib-obinutuzumab; IFN, interferon; IL, interleukin; IRR, infusion-related reaction; MCP, monocyte chemoattractant protein; MIP, macrophage inflammatory protein; TNF, tumor necrosis factor.

**
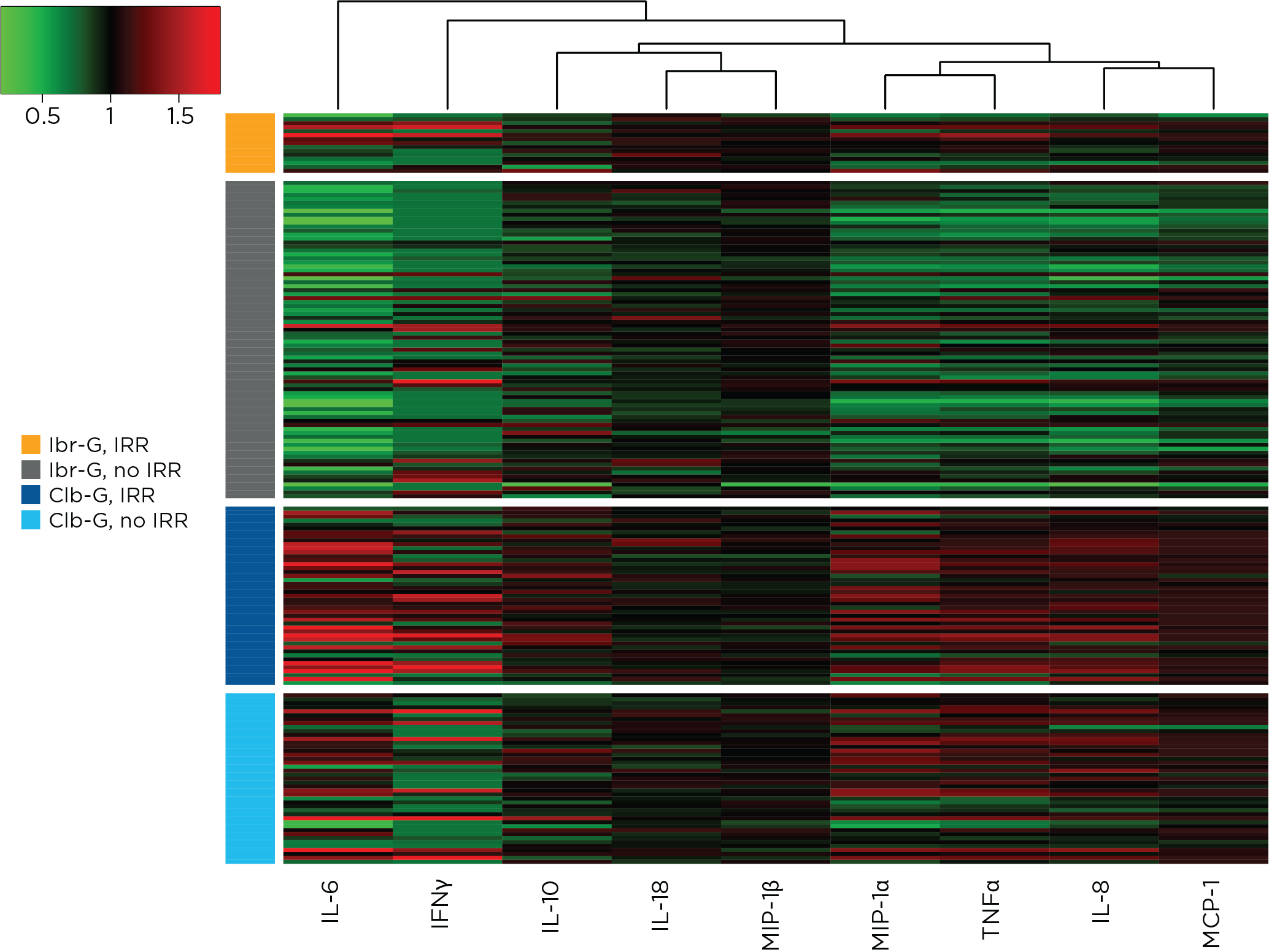
**

**Table S4. Cytokines and Chemokines Associated With IRRs**

| **Cytokine/ chemokine** | **Cellular sources** | **Receptor expression** | **Immune function** |
| --- | --- | --- | --- |
| IFNγ [1] | NK cells, NKT cells,  CD4^+^ Th1 cells, cytotoxic  CD8^+^ T cells | NK cells, DCs, CD4^+^/CD8^+^ T cells | Innate antiviral immunity, adaptive immunity, MHC (class I/II) upregulation, NK-cell activation, Th1 differentiation |
| IL-6 [2,3] | Macrophages, monocytes, endothelial cells, fibroblasts, mesenchymal cells | Hepatocytes, B cells, T cells, fibroblasts, megakaryocytes, hematopoietic stem cells, osteoclasts | Fever induction, acute-phase protein induction, B-cell maturation and antibody secretion, Th17 differentiation, T_reg_ differentiation |
| IL-8/CXCL8 [4] | Monocytes,  macrophages, neutrophils, lymphocytes, endothelial cells, epithelial cells | Neutrophils | CXC chemokine, activation and recruitment of neutrophils |
| IL-10 [5] | CD4^+^ T cells, macrophages, DCs, B cells, NK cells | CD4^+^ T cells, macrophages, DCs, B cells, NK cells | Anti-inflammatory cytokine, T_reg_ survival |
| IL-18 [6] | Macrophages, monocytes, DCs, endothelial cells, epithelial cells | T cells, NK cells, DCs, mast cells, basophils | IFNγ induction, Th1/Th2 differentiation, NK activation, mast-cell activation, basophil activation |
| TNFα [7,8] | Monocytes, macrophages, NK cells, T cells, neutrophils, mast cells, eosinophils, endothelial cells, fibroblasts | T cells, fibroblasts, endothelial cells, epithelial cells | Inflammation, acute-phase protein induction, apoptosis, T-cell activation |
| MCP-1/CCL2 [9] | Macrophages, monocytes, endothelial cells, fibroblasts, epithelial cells | Monocytes, NK cells, memory T cells | CC chemokine, monocyte migration, NK-cell migration, memory T-cell migration |
| MIP-1α/ CCL3 [10] | Monocytes, macrophages, T cells, B cells, DCs, neutrophils, granulocytes, epithelial cells, fibroblasts, mesangial cells | Monocytes, T cells, neutrophils, granulocytes, DCs, NK cells, platelets | Leukocyte migration |
| MIP-1β/ CCL4 [10] | T cells, B cells, DCs, NK cells, neutrophils | Monocytes, T cells, neutrophils, granulocytes, DCs, NK cells, platelets | Leukocyte migration |

IFN, interferon; IL, interleukin; IRR, infusion-related reaction; MCP, monocyte chemoattractant protein; MHC, major histocompatibility complex; MIP, macrophage inflammatory protein; NK, natural killer; Th, T helper; TNF, tumor necrosis factor; T_reg_, regulatory T cells.

**SUPPLEMENTAL REFERENCES**

1. Schoenborn JR, Wilson CB (2007) Regulation of interferon-gamma during innate and adaptive immune responses. Adv Immunol 96:41-101. doi:10.1016/S0065-2776(07)96002-2

2. Tanaka T, Kishimoto T (2014) The biology and medical implications of interleukin-6. Cancer Immunol Res 2 (4):288-294. doi:10.1158/2326-6066.CIR-14-0022

3. Tanaka T, Narazaki M, Kishimoto T (2014) IL-6 in inflammation, immunity, and disease. Cold Spring Harb Perspect Biol 6 (10):a016295. doi:10.1101/cshperspect.a016295

4. van Eeden SF, Terashima T (2000) Interleukin 8 (IL-8) and the release of leukocytes from the bone marrow. Leuk Lymphoma 37 (3-4):259-271. doi:10.3109/10428190009089427

5. Saraiva M, O'Garra A (2010) The regulation of IL-10 production by immune cells. Nat Rev Immunol 10 (3):170-181. doi:10.1038/nri2711

6. Kaplanski G (2018) Interleukin-18: Biological properties and role in disease pathogenesis. Immunol Rev 281 (1):138-153. doi:10.1111/imr.12616

7. Sedger LM, McDermott MF (2014) TNF and TNF-receptors: From mediators of cell death and inflammation to therapeutic giants - past, present and future. Cytokine Growth Factor Rev 25 (4):453-472. doi:10.1016/j.cytogfr.2014.07.016

8. Mehta AK, Gracias DT, Croft M (2018) TNF activity and T cells. Cytokine 101:14-18. doi:10.1016/j.cyto.2016.08.003

9. Deshmane SL, Kremlev S, Amini S, Sawaya BE (2009) Monocyte chemoattractant protein-1 (MCP-1): an overview. J Interferon Cytokine Res 29 (6):313-326. doi:10.1089/jir.2008.0027

10. Menten P, Wuyts A, Van Damme J (2002) Macrophage inflammatory protein-1. Cytokine Growth Factor Rev 13 (6):455-481. doi:10.1016/s1359-6101(02)00045-x
